# Inappropriate Implantable Cardioverter-Defibrillation Detection of Ventricular Fibrillation Induced by Transcutaneous Electrical Nerve Stimulation

**DOI:** 10.19102/icrm.2022.130506

**Published:** 2022-05-15

**Authors:** Shireen Mohammad, Carrie Mayer, Melanie Foisy, Min-Shien Chen, Syamkumar Divakaramenon, Adrian Baranchuk

**Affiliations:** ^1^Division of Cardiology, Queen’s University, Kingston, Ontario, Canada; ^2^Arrhythmia Services, Division of Cardiology, McMaster University, Hamilton, Ontario, Canada

**Keywords:** Electromagnetic interference, ICD, transcutaneous electrical nerve stimulation

## Abstract

We describe 2 cases of electromagnetic interference (EMI) with a transcutaneous electrical nerve stimulation (TENS) device in patients with implantable cardioverter-defibrillators (ICDs). Both patients were using DR-HO’S^®^ pain therapy system for chronic back pain (VGH Solutions Inc., Markham, ON, Canada). In both cases, EMI was inappropriately labeled as ventricular fibrillation. In the first case, the noise detected was of a short duration and did not fulfill the discriminator criteria to deliver ICD therapy. In the second case, inappropriate anti-tachycardia pacing and shocks resulting from EMI were delivered. Both patients were advised not to use TENS devices at home. Increased awareness of EMI resulting in inappropriate ICD therapies using these devices is needed.

## Introduction

Transcutaneous electrical nerve stimulation (TENS) is a commonly used treatment for the relief of acute and chronic musculoskeletal pain.^1^ It involves placing electrodes on the skin, through which TENS is applied at varying frequencies, intensities, and pulse durations of stimulation to maximize pain relief.^1^ Electrical nerve stimulation can create electromagnetic interference (EMI) with the sensing function of pacemakers, implantable cardioverter-defibrillators (ICDs), and cardiac resynchronization therapy devices,^2,3^ resulting in potential inappropriate therapies.^3^

## Case presentations

### Case #1

**Figure 1:**
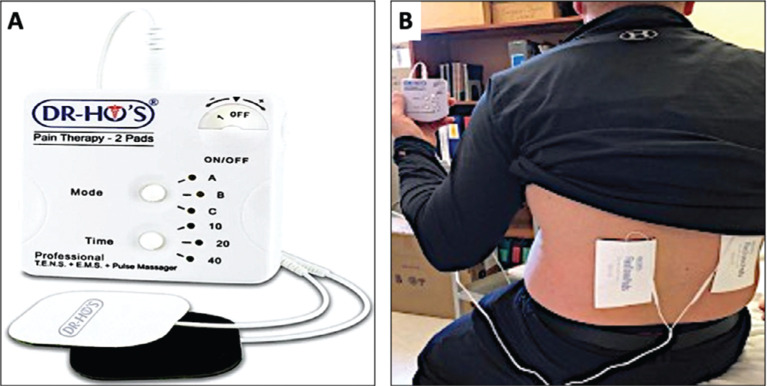
**A:** The transcutaneous electrical nerve stimulation device. (Dr. Ho’s pain therapy system 2-Pad). **B:** TENS device with 2 pads applied to the patient’s lower back for pain control.

**Figure 2:**
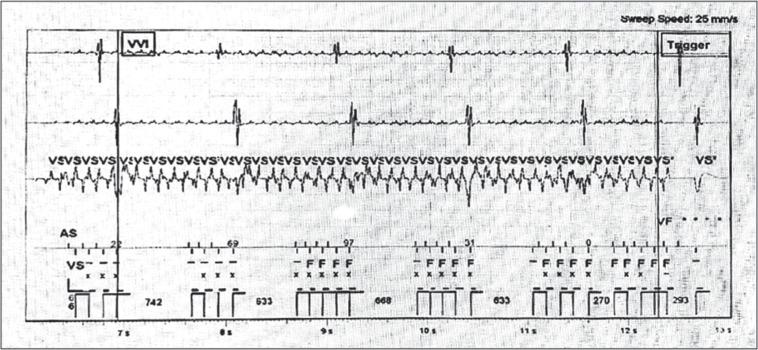
Implantable cardioverter-defibrillator electrogram demonstrating electromagnetic interference misinterpreted as a ventricular fibrillation episode not fulfilling the criteria for therapy.

A 48-year-old man with a history of coronary artery disease and a dual-chamber ICD (2259-40 Q Fortify Assura; Abbott, Chicago, IL, USA) implanted in 2014 due to out-of-hospital cardiac arrest (ventricular fibrillation [VF] terminated with 2 external shocks) came to our clinic for regular follow-up. The patient reported using a TENS device (DR-HO’S^®^ pain therapy system 2-Pad, TENS device; VGH Solutions Inc., Markham, ON, Canada) for chronic lower back pain, including electrical muscle stimulation with a high frequency at 90–140 Hz as shown in **Figures 1A and 1B**. ICD interrogation demonstrated episodes of noise in the right ventricular (RV) lead inappropriately detected as VF **(Figure 2)**. RV sensitivity was programmed at 0.5 mV. Noise was non-sustained and did not fulfill the criteria for the ICD to deliver therapy. The patient was advised not to use the TENS device to avoid inappropriate shocks. No further episodes of RV noise were detected during follow-up.

### Case #2

**Figure 3:**
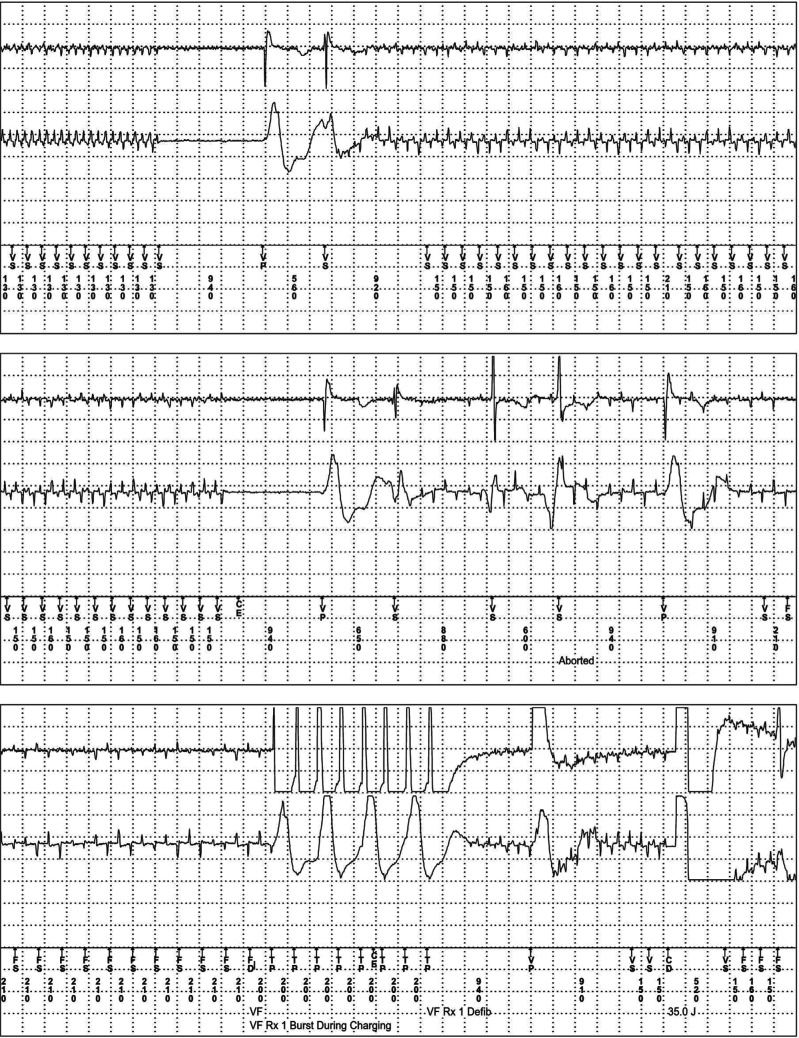
Implantable cardioverter-defibrillator (ICD) electrogram showing erroneous interpretation of ventricular fibrillation by the ICD, which leads to pacing-inhibition and inappropriate therapies.

An 84-year-old man with a single-chamber ICD (Medtronic Protecta, Minneapolis, MN, USA) implanted in 2014 for complete atrioventricular block against the background of ischemic cardiomyopathy and cardiac amyloidosis presented to the clinic after 4 consecutive shocks while he was using a DR-HO’S^®^ TENS device for his chronic back pain. He was pacing-dependent. ICD interrogation showed regular, high-frequency, low-amplitude noise interference **(Figure 3)**. RV sensitivity was programmed at 0.3 mV. There were 3 different frequencies of noise detected by the device, which were all erroneously interpreted as VF. The noise also inappropriately inhibited ventricular pacing. It eventually led to 5 inappropriate therapies (1 anti-tachycardia pacing [ATP] and 4 shocks). The patient switched off the TENS device just before the onset of the fourth shock, which evolved into a committed shock. The patient was informed about this interference and advised against further use of the TENS device. Further follow-up showed no RV lead noise.

## Discussion

EMI still represents a challenge to the sensing function of pacemakers and ICDs. Such a challenge takes place in both medical and non-medical environments.^4^ TENS and spinal cord stimulators may result in inappropriate inhibition for pacing or false tachyarrhythmia detection, resulting in ICD therapies (ATP and/or shocks). Other potential ICD responses to TENS include high atrial tracking in dual-chamber pacemakers.^3^ There is a possibility for microprocessor reset in extreme cases.^4^ Despite major advances in cardiac electronic devices’ technology, EMI continues to be a frequent problem in device clinics. Several reports from prior publications can be found in the literature.^1,2,5^ This interference with proper device functioning compromises precise diagnostic and therapeutic function of cardiac devices.^2,5^ The cases presented in this series illustrate the risk of EMI in triggering unnecessary ICD therapies. Modern pacing systems have evolved in various ways to decrease susceptibility to potential EMI adverse events.^1–4^ Bipolar leads were developed to reduce the “antenna” effect and the oversensing phenomenon of external electromagnetic signals by incorporating hermetically sealed titanium shield housing or stainless-steel cases surrounding circuitries specially designed with band-pass filters. This allows signals outside the narrow range of cardiac depolarization frequencies (10–50 Hz) to be filted out.^1–4^ The risk of TENS interference seems to be lower when electrodes are placed further away from the device, eg, the lower limbs for drop foot,^2–6^ and when a lower frequency is used.^6^ However, as illustrated by these 2 cases, existing TENS devices can still interfere with the normal functioning of ICDs, with deleterious (and potentially serious) consequences for the patient. The ideal scenario would be to deliver TENS in a monitored setting, with ICD therapies temporarily turned off, to facilitate the treatment required. However, this approach may not be always feasible, and restrictions to the use of TENS may apply.^1^

## Conclusion

Technology for the protection and insulation of pacemakers and ICDs has significantly improved; however, adverse events related to EMI still occur. We recommend that patients with ICDs not use TENS devices at home.
